# Supplementary material for: Left Prefrontal tDCS during Learning Does Not Enhance Subsequent Verbal Episodic Memory in Young Adults: Results from Two Double-Blind and Sham-Controlled Experiments
Source: Brain Sci. 2023 Jan 31;13(2):241. doi: 10.3390/brainsci13020241 (PMC9954521; doi:10.3390/brainsci13020241)
Supplement: Supplementary file 1 [file brainsci-13-00241-s001.zip › brainsci-2124755-supplementary.pdf]

Supplementary materials

Survey of sensations related to transcranial electrical stimulation

This questionnaire (for the original, see Fertonani et al., 2015) requires participants to report to what degree they have experienced potential discomfort and side-effects of stimulation: 1) Itching, 2) Pain, 3) Burning, 4) Warmth/Heat, 5) Pinching, 6) Metallic/Iron taste, 7) Fatigue, 8) Other.

Participants answered the following question for each item on a Likert-scale:

'Did you experience any discomfort or annoyance during the electrical stimulation? Please answer the following questions regarding the different sensations and indicate the degree of intensity of your discomfort according to the following scale:'

The answer options are: None = I did not feel the described sensation (0), Mild = I mildly felt the described sensation (1), Moderate = I felt the described sensation (2), Considerable = I felt the described sensation to a considerable degree (3), Strong = I strongly felt the described sensation (4).

Total scores for each participant were calculated using the 0-4 key above.

Edinburgh Handedness Questionnaire

Participants were pre-screened for handedness and completed the Edinburgh handedness Inventory test (Oldfield, 1971) in order to determine degree of right-handedness. This required self-rating their preferred hand use on (right or left) on list of commonly performed everyday tasks. These were: 1. Writing 2. Drawing 3. Throwing 4. Scissors 5. Toothbrush 6. Knife (without fork) 7. Spoon 8. Broom (upper hand) 9. Striking Match (match) 10. Opening box (lid).

Participants were required to indicate whether they have a preferred hand (one tick) or could only do a task with a particular hand (two ticks). For any tasks where they did not have a preferred hand, both left and right columns were required to be ticked. Summary scores were calculated from number of ticks for each hand as follows: 100 * ((Right – Left) / (Right + Left)). Positive scores indicate right handedness.

Word lists: Experiment 1

The length of necessary word lists for the recognition test was piloted with volunteers to help determine an optimal task length to avoid floor or ceiling effects. Based on this pilot data, a list length of 180 was selected for the encoding task. This allowed for at least 20 forgotten items (typically 20-40) in pilot participants during same day retrieval tests, thus helping to avoid ceiling effects.

Words comprising stimuli sets were selected from the MRC Psycholinguistic Database (Coltheart, 1981). Words with high levels of concreteness (>400) and imageability (>400), with a length of 3-10 phonemes were selected. Counterbalancing was applied in order to ensure that words had similar written frequency and length when presented either as a target or a non-target, or used in the recognition test on either Day 1 or Day 2.

Word lists: Experiment 2

The required length of word lists for the recall test was determined following piloting the experimental tasks. Pilot participants typically remembered 15-20 items of the 40 targets learned on Day 1, i.e. this length of the stimuli set allowed avoiding floor- or ceiling effects.

Words of comparable length (2-3 syllables, 6-10 letters) with high levels of imageability (>450), concreteness (>438) and frequency (>40) were selected from the MRC Psycholinguistic Database (Coltheart, 1981). Selected items were allocated into counterbalanced groups with similar

characteristics along these attributes, to ensure similarity of characteristics of target and non-target words.
